# Trends in mass spectrometry imaging for cardiovascular diseases

**DOI:** 10.1007/s00216-019-01780-8

**Published:** 2019-04-12

**Authors:** Stephanie T. P. Mezger, Alma M. A. Mingels, Otto Bekers, Berta Cillero-Pastor, Ron M. A. Heeren

**Affiliations:** 1 Maastricht MultiModal Molecular Imaging (M4I) Institute, Division of Imaging Mass Spectrometry, Maastricht University, Universiteitssingel 50, 6229 ER Maastricht, The Netherlands; 2 Central Diagnostic Laboratory, Maastricht University Medical Center, P.O. Box 5800, 6202 AZ Maastricht, The Netherlands; 3 CARIM School for Cardiovascular Diseases, Maastricht University, Universiteitssingel 50, 6229 ER Maastricht, The Netherlands

**Keywords:** Mass spectrometry imaging, Cardiovascular diseases, MALDI, SIMS, Lipids, Proteins

## Abstract

Mass spectrometry imaging (MSI) is a widely established technology; however, in the cardiovascular research field, its use is still emerging. The technique has the advantage of analyzing multiple molecules without prior knowledge while maintaining the relation with tissue morphology. Particularly, MALDI-based approaches have been applied to obtain in-depth knowledge of cardiac (dys)function. Here, we discuss the different aspects of the MSI protocols, from sample handling to instrumentation used in cardiovascular research, and critically evaluate these methods. The trend towards structural lipid analysis, identification, and “top-down” protein MSI shows the potential for implementation in (pre)clinical research and complementing the diagnostic tests. Moreover, new insights into disease progression are expected and thereby contribute to the understanding of underlying mechanisms related to cardiovascular diseases.

**Electronic supplementary material:**

The online version of this article (10.1007/s00216-019-01780-8) contains supplementary material, which is available to authorized users.

## Cardiovascular diseases and clinical diagnosis

For the last 15 years, the leading cause of death worldwide is cardiovascular diseases, in particular ischemic heart disease and stroke [1]. During such an acute ischemic event, an atherosclerotic plaque in the blood vessels is ruptured, causing obstructive blood flow and a lack in oxygen supply in the surrounding tissue. The build-up of this plaque in the innermost layer of the artery wall is the result of narrowing of the artery and the loss of arterial elasticity. It is a complex process with many different factors, which are not completely understood yet, and is also related to other chronic diseases of the heart and blood vessels [2].

Cardiovascular diseases are diagnosed using a range of clinical tests, from laboratory to imaging-based analyses. Laboratory tests check for general blood components like lipids (fats, cholesterol) or for cardiac specific biomarkers, like cardiac troponins and natriuretic peptides. Imaging techniques check for structural and spatial information, either invasively or non-invasively, being often echocardiography, cardiac MRI, or computed tomography [3]. Very important considerations in the diagnostic work-up are also patient medical record, family history, and risk factors. The diagnosis of acute myocardial infarction (MI) is based on typical clinical signs and an electrocardiogram. In case of non-ST elevation MI the additional detection of a rise and/or fall of cardiac biomarkers (either cardiac troponin T and I) or other imaging are required [4]. The clinical tests and imaging techniques are however not sufficient to obtain all molecular information on a spatial level.

In cardiovascular research, on the contrary, far more sophisticated techniques such as mass spectrometry imaging (MSI) are available to obtain more in-depth information on the involved components and pathways. The characterization of these biochemical changes provides information on the pathophysiology which may eventually be used in clinical applications [5, 6]. MSI is an emerging tool and obtains spatial information of multiple molecules without prior knowledge; therefore, this might be an interesting complementary tool as compared with current clinical methodologies, for instance, immunohistochemistry in the field of tissue characterization.

In this article, we provide an overview of available MSI protocols and applications in cardiovascular research (see Electronic Supplementary Material (ESM) Table S1), with a focus on the following molecular classes: lipids, proteins/peptides, and metabolites. We will elaborate on the choices to be made, from sample handling to instrumentation. Some of these choices depend on the molecular class of interest, from washing steps, enzymatic digestion, matrices, to mass range. Finally, we discuss the trends in MSI for cardiovascular diseases with an outlook to future work.

## Mass spectrometry imaging: a brief introduction

**Fig. 1:**
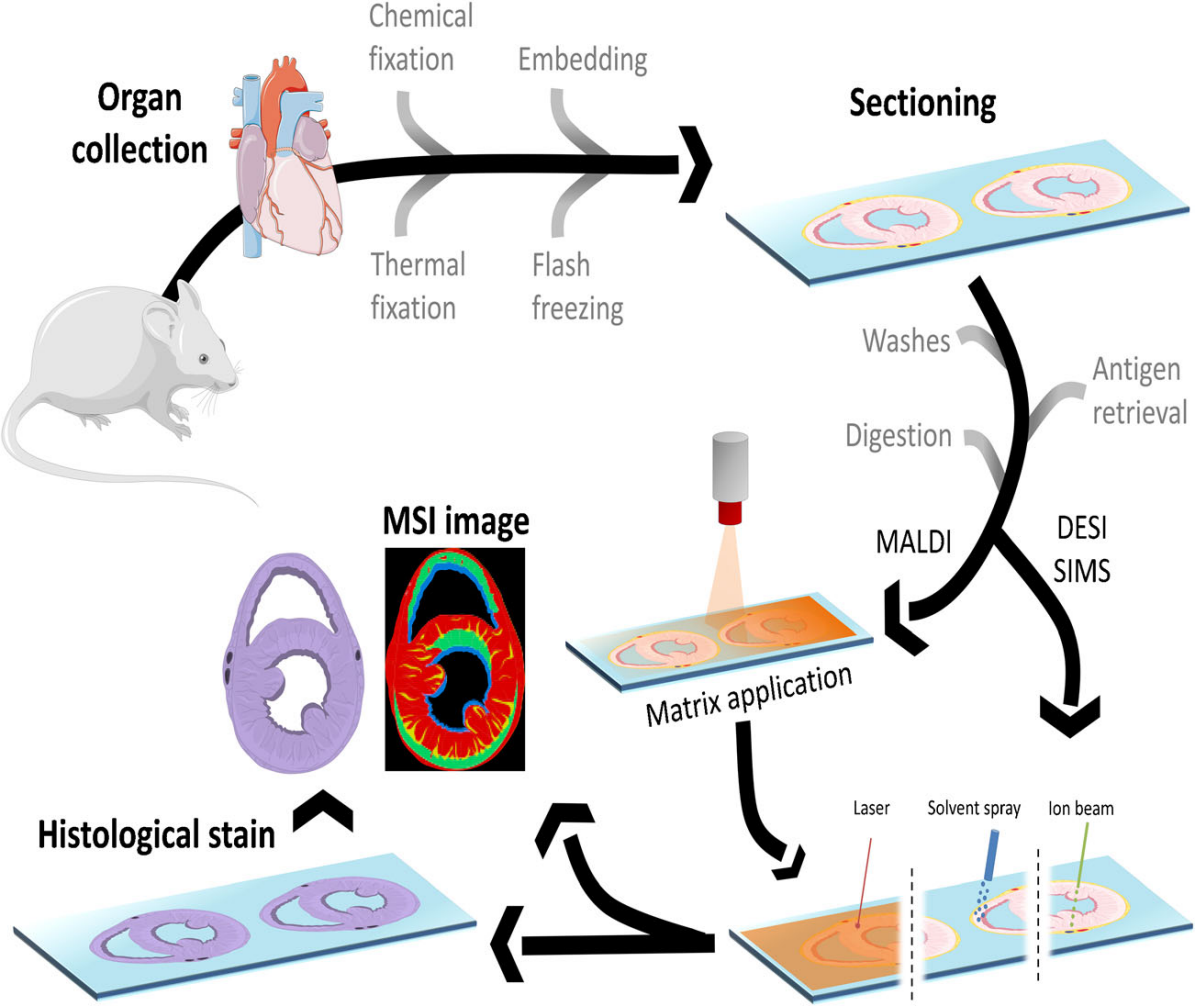
Schematic overview of the workflow for MSI. First, the organ is harvested, then either snap-frozen or fixed, with or without embedding. Next, the tissue is cut and mounted on a slide. Then, optional and depending on the analyte of choice, washing steps, antigen retrieval, and digestion protocols are performed. For MALDI, a matrix is applied on top of the tissue, and a laser beam generates the ions for detection. For SIMS, an ion beam generates the secondary ions for detection. For DESI, a charged solvent extracts the ions from the tissue. Afterwards, a histological stain can still be done

In a typical MSI experiment, a mass spectrum is generated for every pixel on the tissue section and information on the molecular content is captured (Fig. 1). The two most frequently used ionization techniques in cardiovascular MSI are matrix-assisted laser desorption/ionization (MALDI) and secondary ion mass spectrometry (SIMS). In *MALDI*, the analyte molecules are extracted from the tissue section and incorporated into matrix crystals. Irradiation of the matrix-covered sample with a laser beam (typically a pulsed UV laser) then produces gas-phase molecular ions that are mass separated in a mass spectrometer. After ion detection, a mass spectrum is generated. This process recurs on each individual analysis position and a collection of mass spectra is acquired. This dataset can be transformed into an image for each individual molecule present in the acquired data. A histological stain can be performed following the MSI experiment and co-registered with this MSI data with respect to the tissue pathology. In that case, the matrix coating needs to be removed prior to the histological staining procedure.

In *SIMS*, a high-energy primary ion beam is used to bombard the tissue surface, which leads to the emission of atomic, fragmented, and molecular (the so-called secondary) ions which are subsequently analyzed in the mass spectrometer [7, 8]. Currently, the development of ambient molecular imaging techniques, such as desorption electrospray ionization (DESI), is emerging. In *DESI*, charged droplets impact the surface and extract analytes from the tissue surface. Ions are generated through electrospray ionization following the droplet interaction with the surface. This technique does not require high vacuum and can be deployed for samples that are not vacuum compatible [9]. SIMS and DESI both are often performed without extensive sample preparation on untreated tissue sections.

## Mass spectrometry imaging in cardiovascular diseases

The first and crucial aspect for MSI is *sample handling* after tissue collection. It is very important to store the tissue in an appropriate manner to preserve structural information, and halt biological processes. Ischemia of tissue can very quickly result in molecular changes that will affect the MSI results. In pathology, the maintenance of the correct spatial information, reduction of degradation, and delocalization are routinely done by formalin-fixed paraffin embedding (FFPE). However, FFPE tissue is less compatible with MSI, due to the cross-linking caused by formalin, making ionization and identification difficult [10]. Developments in sample preparation have improved MSI possibilities for the use of FFPE samples for metabolite and protein/peptide imaging [11–15]. The preferred tissue preservation method for MSI that provides access to a wide variety of molecular classes is still cryo-preservation through immediate flash freezing after tissue resection/collection.

The next step is *tissue sectioning*, and for frozen tissue, a cryo-microtome is used. Operating temperature ranges from − 5 to − 25 °C and depends on the tissue [16]. For frozen arterial and cardiac tissue, temperatures between − 20 and − 25 °C have been used [16, 17]. The typical section thickness varies between 10 and 20 μm to avoid cracking or excessive drying times. Fragile cardiovascular tissue can be supported during sectioning with an embedding medium, for example, gelatin [18, 19] or carboxymethyl cellulose [20, 21]. The use of optimal cutting temperature (OCT) compound is not recommended due to possible polymeric contamination of the MS spectra [22] that obscure some of the relevant molecular details. Nevertheless, OCT has been used in some cardiovascular disease studies [17, 23–25]. Tissue sections are then thaw-mounted on glass slides or electrically conductive indium tin oxide (ITO)–coated glass slides, for orthogonal and non-orthogonal instruments, respectively. A microtome is used for FFPE material, and the section thickness varies between 3 and 15 μm [12, 13, 26]. Before further sample preparation, the FFPE sample needs to undergo deparaffinization and when interested in peptide/protein analysis, also antigen retrieval.

It has been shown to be beneficial to add an adhesive substance to the glass slide before mounting the tissue, in cardiovascular studies poly l-lysine is used [17, 27]. This prevents tissue loss during extensive wash protocols.

Depending on the analyte of interest, the protocol can include washing and/or digestion steps. *Washing steps* reduce ion suppression by the removal of salts, small molecules, and/or lipids [28, 29]. Therefore, these steps are used for protein/peptide and in general not for lipid and or metabolite imaging. *Digestion steps* are included to reduce the size of larger biomacromolecules such as proteins and glycans and make their fragments (proteolytic peptides and oligosaccharides) amenable to MSI.

*The application of a matrix* layer on top of the tissue is the next step for MALDI-MSI. This matrix solution consists of an organic acid (the matrix) and an organic solvent, and in some cases, trifluoroacetic acid is added to assist in protonation of the analytes. The organic acid forms analyte-matrix crystals, which absorb the energy from the laser, enabling the ionization and acting as a proton source [21]. Different methods are available to ensure the formation of a uniform matrix layer on top of the tissue surface, namely spraying [22], vibrational vaporization [23], nebulization [24], and sublimation [27, 30]. These methods generate layers of matrix crystals of different sizes and thickness. In the case of metal-assisted SIMS, a thin layer of gold can be deposited on the sample to improve molecular ion yield [31]. The attainable spatial resolution in matrix-based MSI strategies is determined by the matrix crystal size and the laser spot size. The narrow width of the ion beam and the reduced sample preparation in SIMS result in a spatial resolution that outperforms MALDI by one to two orders of magnitude. The spatial resolution in DESI-based MSI is influenced by the size, shape, stability, and solvent used for the spray. It can be beneficial to perform reactive DESI for some endogenous compounds with low ionization efficiencies. In this approach, the analyte is derivatized with a reagent in the spray solvent [20, 32].

The mass analyzer used for MSI defines the mass accuracy, mass resolution, and resolving power. For MALDI-MSI, the most frequently used mass analyzer is the time-of-flight (TOF), which has a theoretical unlimited mass-to-charge (*m*/*z*) range and single ion detection capabilities [33]. A TOF or even TOF-TOF has often been used for the analysis of different molecular classes in cardiovascular studies [17, 23, 27, 34, 35]. MALDI can also be combined with a Fourier transform ion cyclotron mass spectrometer (FT-ICR) [17], which provides higher mass accuracy and mass resolution compared with a TOF-MS; however, this instrument is less suitable for high *m*/*z* analysis. Other optional mass analysers are a quadrupole TOF [35], a LTQ XL linear ion trap [36], and an orbitrap Fourier transform MS (FTMS) [37]. For SIMS MSI, a TOF-based mass spectrometer is most often used [19, 25, 31, 38–40]. An ion source, for instance, gold or bismuth liquid metal ion gun, generates a pulse of primary ions that are accelerated towards the surface. For DESI-MSI, the source can be coupled with different mass analyzers that can cope with continuous beams, such as a triple quadrupole, q-TOF, FT-ICR, and orbitrap. In addition to the aforementioned protocols, tandem MS is also applied for identification of particular compounds. The generated fragments in tandem MS are compared with database libraries and used for the identification of the molecule; tandem MS is required for a proper structural identification. When using accurate mass analysis, the intact mass is used to determine the composition, database matching, and identification. For example, in lipid identification, high-resolution mass analysis enables a confident assignment of lipid classes; tandem MS is required for the identification of the individual fatty acid (FA) chains. Structural lipid identification will increase the understanding in lipid biochemistry and their roles in (patho)physiological processes.

## Lipid MSI applied to cardiovascular diseases

Lipids play a vital role in many cellular processes. It is a diverse and complex class of molecules with high structural variability. It includes among others FA, steroids and many classes of phospholipids (PL). The heart uses FA as its major energy substrate, and besides FA, its lipidome consists mainly of (lyso)phospholipid, sphingolipid species, and neutral lipids [41, 42].

For the MSI analysis of specific lipid species, Angel et al. obtained signal enhancement by adding aqueous washing steps prior to matrix deposition [43]. Their protocol using ammonium formate or ammonium acetate showed increased signal intensity in negative ionization mode and reduction of sodium and potassium adducts in positive ionization mode. The *choice of matrix* and the ionization polarity (positive and/or negative) are important for all MSI lipid experiments. The success of a study is affected by the natural polarity of many lipids. When analyzing in positive ionization mode, sphingomyelins (SM), phosphatidylcholines (PC), and neutral lipids, such as triacylglycerols (TAG) and cerebrosides, can be detected [44, 45]. In negative ionization mode, phosphatidylinositols (PI), phosphatidylserines (PS), phosphatidylethanolamines (PE), phosphatidylglycerols (PG), glycosphingolipids, and cardiolipins (CL) are found. For cardiovascular lipid research, 2,5-dihydroxybenzoic acid (DHB) is the most commonly used matrix, as it can be used in both ionization polarities [17, 23, 27, 43]. Other matrices in negative ion mode are 2,6-dihydroxyacetophenone (DHA) and 9-aminoacridine (9AA); in positive ion mode, α-cyano-4hydroxycinnamic acid (CHCA) [23]; and for dual polarity also, 1,5-diaminoaphthalene (DAN) [24].

Cardiovascular lipid imaging was mostly performed with a (Q)TOF or TOF-TOF instrument, coupled with MALDI [23, 27, 34, 46, 47] or SIMS [19, 25, 31, 38–40]. Structural information, high mass accuracy, and identification were done using an FR-ICR, LTQ XL linear ion trap, or orbitrap coupled with MALDI [17, 34, 36, 37, 47] or DESI [20, 32].

**Fig. 2:**
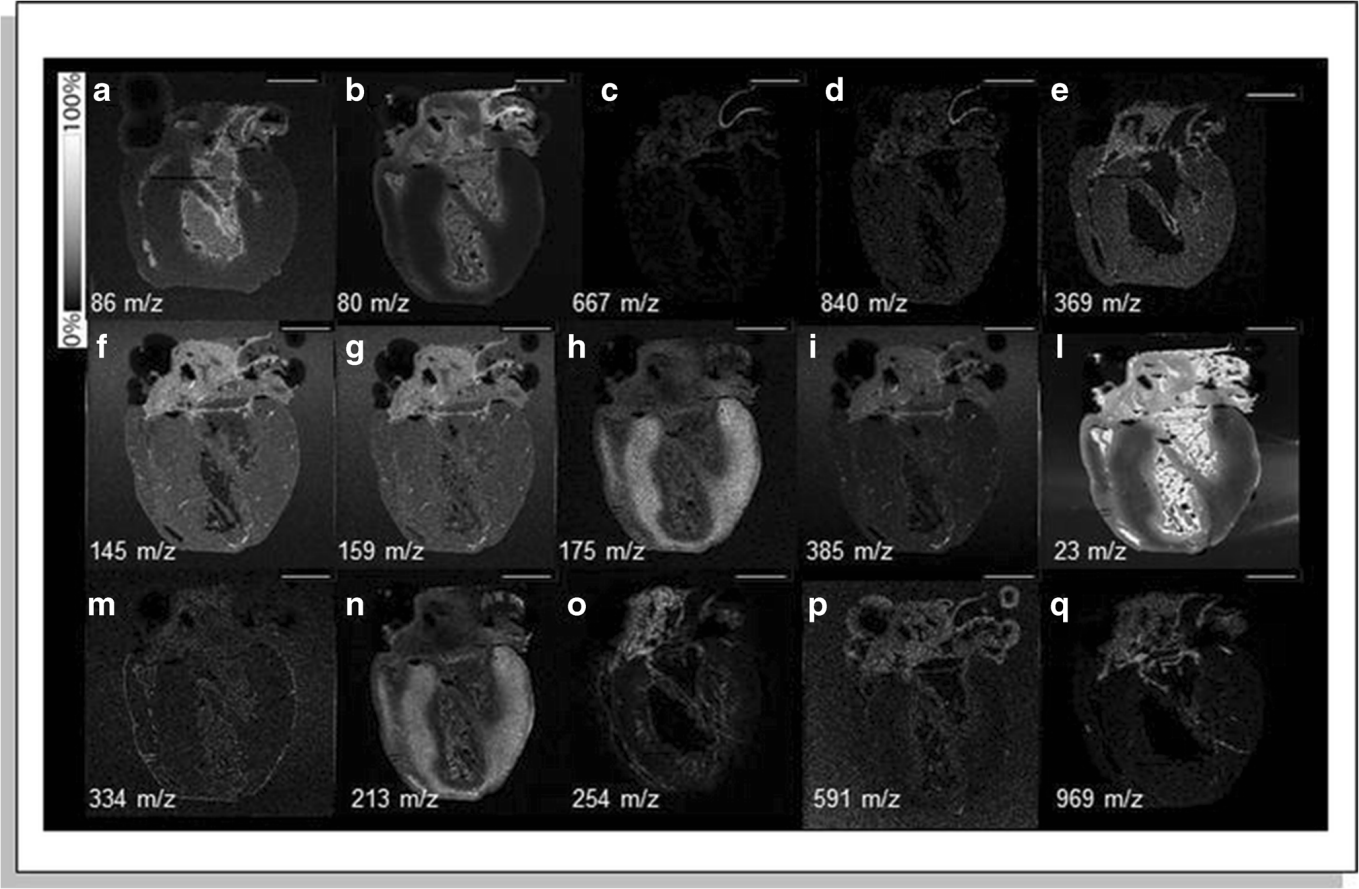
Sagittal sections of a rat hearts imaged using metal-assisted SIMS. Anatomical features are visualized in SIMS ion images, specific for the aorta wall (**a**–**d**), ventricles (**h** and **n**), and pericardium (**m**). Localization of the main cholesterol ions (*m/z* 369 and *m/z* 385) shows localization in the aorta wall, aorta valve, right coronary artery, and right and left atria, image **e** and **i** respectively. Scale bar = 100 μm. Reprinted with permission from Springer Nature Customer Service Centre GmbH: Springer Anal Bioanal Chem. Fornai L, Angelini A, Klinkert I, Giskes F, Kiss A, Eijkel G et al. Three-dimensional molecular reconstruction of rat heart with mass spectrometry imaging. Anal Bioanal Chem. 2012;404(10):2927–38, copyright 2012. [31]

Multiple MALDI-MSI studies focused on the distribution of phospholipid species in healthy cardiac tissue. Lipids play an important role as structural biomolecules and in signal transduction processes. One of these species is cardiolipins, a lipid found in the mitochondrial membrane and closely associated with mitochondrial function. It is found to be homogeneously distributed in the ventricular myocardium of a healthy rat heart [27]. Other lipid species predominantly found in the rat myocardium were PCs and PEs, while the vessel region had a higher abundance of TAG and PI species [37]. Researchers have constructed a 3D MSI model of a rat heart to provide a full view of the cardiac structure and morphology, obtained with metal-assisted TOF-SIMS [31]. Distinctive peaks for the aorta wall, valve, ventricles, atria, pericardium, and endocardium were found (Fig. 2) and used for the reconstruction of the 3D volume. A cross-species validation study revealed similar lipid patterns in rat and mouse hearts, while a human ventricular sample showed distinctive structures, with higher cholesterol in the myocardium, and high diacylglycerol species and ceramide in the pericardium. The distribution of metals, important components in cellular and molecular processes in the heart, was visualized in a mouse heart by combining laser ablation inductively coupled MS (LA-ICPMS) and SIMS [25]. These imaging results suggested higher concentrations of Zn, Mn, Cu, Mg, and Ca in the right compared with the left ventricle. Using MALDI-MSI, other authors found a, as yet unidentified, phospholipid (*m/*z 600) that differentiated the hinge region and valve cusp in mouse aortic valves [46], and in ovine aortic valves, a combination of PC, PE, and SM species was distinctive for the fibrosa and spongiosa regions while this differentiation could not be made for pre-natal valves [17].

Regarding cardiovascular diseases, MALDI-MSI of an infarcted heart revealed a higher ion signal of lysoPLs and a decrease in intact PLs in the infarcted region and supports the hypothesis that phospholipase A_2_ (PLA_2_) and arachidonic acid play a role in MI, with an increased activity of PLA_2_ in the infarcted area [36]. Margulis et al. applied a machine learning algorithm to DESI-MSI data to improve data analysis. This resulted in a molecular signature that distinguished between ischemic and perfused cardiac tissue in mice [32]. The 62 selected molecular ion peaks classified tissue types across pixels with high accuracy and precision; one of the ion peaks was the amino acid taurine, found more abundantly in perfused tissue. Additionally, polyunsaturated long-chain fatty acids were found to be depleted in infarcted areas.

Another frequently studied cardiovascular disease is atherosclerosis; Castro-Perez et al. showed a distinct distribution of lipids across the aortic plaque region, with free cholesterol in the plaque and necrotic core, cholesteryl ester (CE) species in the lesion area, lyso-PC (LPC) species in the arterial wall and atheroma, and PC species homogenously present across the tissue [23]. Others found increased lysolipids, PI, PG, and SM species in the intima, and specific localization of TAG, diacylglycerophosphate (PA), SM, and PE-Cer species in calcified regions of the atherosclerotic samples [34, 47]. Zaima et al. investigated mouse atherosclerotic lesions; they identified PC species characteristic for the smooth muscle cell region and CE species in the lipid-rich region and found one unidentified *m/z* value (566.9) specific for the calcified region [48]. Their analysis of human atherosclerotic lesions showed similar distributions for PC and CE species compared with mouse, with the addition of a TAG in the lipid region and a different *m/z* value (539.0) for the calcified region. A 3D MSI reconstruction of atherosclerotic plaques from mouse heart and human carotid by Patterson et al. revealed similar lipid patterns [24]. Both contained LPCs in the bulk of the plaque, and PC species in the plaque interior wall. In addition, TAGs were present in the mouse aortic valve cusps and ceramide species showed co-localization with LPCs in the human plaque. Silver-assisted LDI MSI of the mouse plaque displayed free FAs containing unsaturated FA chains co-localized with PCs and specific localization of cholesterol not co-localized with free FAs. Interestingly, Tanaka et al. focused on the role of LPC acyltransferase-3 (LPCAT3) and suggested a relation in LPCAT3 expression and atherosclerosis progression as they observed an increase of LPC and a decrease of arachidonyl-PC species [49]. The lipid metabolism in a human plaque was investigated using TOF-SIMS and showed different distributions of several FA species, cholesterol, vitamin E, PA, PC species, SM, and PI fragments in the atherosclerotic intima and medial layer [38, 40]. The human atherosclerotic plaque imaged with DESI-MSI showed lipid-rich regions across the plaque, containing different compositions of cholesterol, SM, and PC species. In addition, DESI-MS identified LPC species in the plaque that are known to be more abundant in oxidized LDL found in plaques [20].

Lipid analysis of a vascular graft that was removed from a human body, containing a plaque-like occlusion, revealed the deposition of cholesterol, SM, and PC species [50]. A comparison between this graft and other research on atherosclerotic arterial tissue showed a remarkable resemblance in the lipid content, not only in the plaque but also in the artificial vessel.

## Application of peptide and protein MSI to cardiovascular diseases

Proteins and peptides are fundamental components of cells and play important roles in many biological processes. Protein imaging can be done “bottom-up” or “top-down,” detecting proteolytic peptides or intact proteins respectively. Peptides are generated by enzymatic or chemical digestion, a conventional approach in proteomics, measured and identified by a combination of high-resolution MS, tandem MS–based peptide sequencing, and database searching. The *bottom-up protein imaging* approach is often used when only FFPE material is available; however, a wide range of tissues can be used. FFPE tissue requires dewaxing and antigen retrieval protocols to make the tissue proteins amenable for enzymatic digestion.

On the contrary, *top-down protein imaging* does not involve any enzymatic digestion. The intact protein is desorbed and ionized from the matrix covered tissue surface followed by controlled fragmentation inside the mass spectrometer using CID, ECD, UVD, or a combination thereof. Unlike bottom-up, this approach potentially allows full sequence coverage, aims at the preservation of existing post translational modifications, and provides detailed structural information. For intact protein in MALDI-MSI, the use of fresh frozen tissue is favorable, as formalin-fixed tissues require additional processing and washing steps. Fixation of the protein content with Carnoy’s solution or acidified ethanol [26] can be beneficial; another approach that works well is ethanol-preserved paraffin embedding [51].

In protein MSI, washing steps are crucial for the removal of salts, metabolites, and lipids to prevent signal suppression. The most common washing solution is ethanol. For enzymatic digestion, the enzyme is applied to the tissue and digestion is enabled in a high-humidity chamber. In cardiovascular research, the most frequently used enzyme, and the gold standard in proteomics, is trypsin, which has specific cleavage sites at the C-terminus of lysine or arginine. Other enzymes used are chymotrypsin, pepsin, elastase, recombinant LysN, PNGaseF, LysC, ArgC, AspN, or GluC, all with their own specific cleavage sites [52]. Chemical digestion involves the treatment of the sample with reactive solutions containing, for example, formic acid, hydrochloric acid, or chemicals such as cyanogen bromide. This approach leads to high-mass peptides, suitable for middle-down proteomics [53–55].

Commonly used *matrices* for peptide and protein MSI are DHB, CHCA, and sinapinic acid (SA) [12, 13, 26, 28, 34, 47, 56–59]. A MALDI TOF-TOF is the most frequently used *instrument* for peptide and protein MSI for cardiovascular diseases, operated in positive ion mode with a range up to 30 kDa [12, 13, 26, 28, 34, 47, 56, 60]. Linear mode was mostly used to enhance the signal of high-mass molecules, while the reflectron mode was used for small proteins and peptides. Furthermore, validation and identification were conducted using tandem MS and accurate mass MS, with an FT-ICR, orbitrap, or quadrupole ion trap TOF. Additional (nano) LC-MS/MS measurements are performed for the identification of proteins.

MSI has been used in several studies into peptides and protein; for example, a baseline measurement of the chick’s cardiac proteome was established by Grey et al. who combined the MALDI-MSI data obtained with different matrices and observed a distinct spatial distribution for different combined protein signals [26]. Identification of these signals was not performed; however, they suggest the use of the established methodology in future experiments for the characterization of heart development stages in healthy and diseased hearts.

**Fig. 3:**
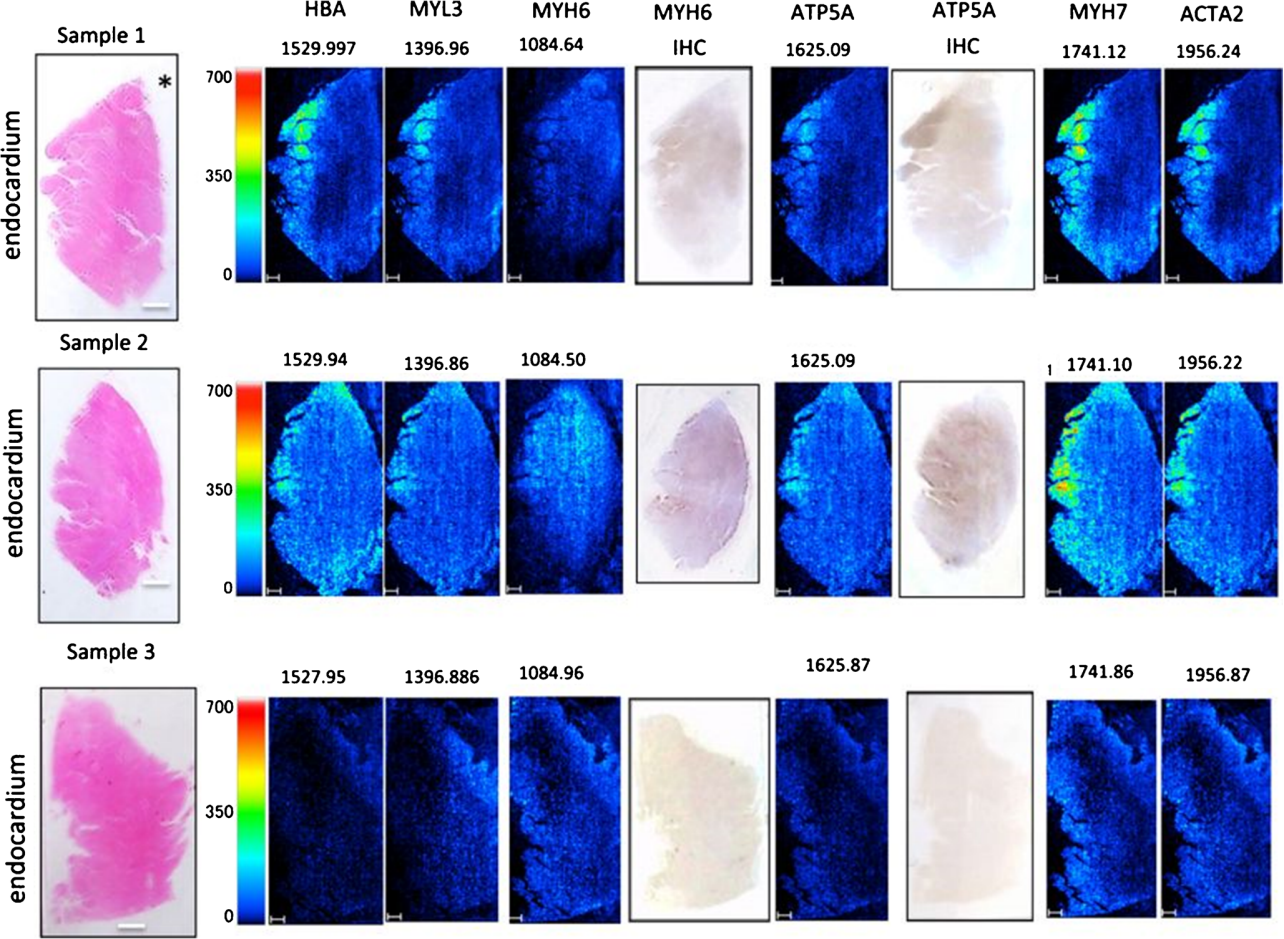
Peptide MALDI-MSI analysis of cardiac tissue. The left column contains H&E staining and orientation (endocardium and pericardium) of the samples. Other columns contain ion images of the identified proteins including *m/z* values or the corresponding immunohistochemistry as indicated above the images. The “*” represents the coronary artery. [12]

The role of different peptides and proteins in *myocardial infarction* (MI) has been studied in a mouse MI model [28]. The spatial distribution of the tyrosine kinase receptor ephrinA1, both as intact protein and tryptic peptides, was investigated by Lefcoski et al. and revealed a higher expression of ephrinA1 fragments in healthy compared with MI mouse hearts. Within the MI heart, three regions (injured, border, and remote) were identified to have specific protein signatures. Interestingly, the injured region showed proteins involved in redox processes, mitochondrial and metabolic enzymes, lipoproteins, and phagocytic vesicles. The results suggesting the involvement of mitochondrial enzymes are in line with the lipid findings of Menger et al. [36]. The remote region displayed proteins indicating increased remodeling, while data from the border region suggested high transcription, translational activity, and defense response. Furthermore, Alghamri et al. investigated the role of the enzyme aminopeptidase A (APA) in the metabolism of cardiac angiotensin (ANG), focussing on ANG II and ANG-(1-7) [60]. This metabolism pathway was visualized using a MALDI-MSI enzyme assay. In this approach, the heart sections were incubated with ANG II. Higher APA protein levels were detected in MI compared with sham mouse hearts, suggesting the degradation of ANG-(1-7) in cardiac repair and reduction of the ventricular function after MI. Also, human MI cardiac tissue was used to identify proteins reflecting cardiomyocyte viability [12]. The damaged lesions showed an enhanced signal for hemoglobin subunit α, adenosine triphosphate synthase subunit alpha (ATPA), and the sarcomeric proteins myosin-6, myosin-7, myosin light chain 3 (MYL3), and alpha actin 2 (Fig. 3). Validation of the results for MYH6 and ATPA was done with immunohistochemistry.

Next, a distinct peptide profile was found for the differentiation of three *atrial fibrillation* subtypes (paroxysmal, persistent, and long-lasting persistent) [13]. MALDI-MSI of human left atrial appendage specimens showed a discriminative distribution of ATPA, alpha 1 type I collagen, MYL4, histone H1.3, neuroblast differentiation-associated protein, cadherin-13, and vimentin in the myocardial tissue.

In *atherosclerotic tissue*, a significant alteration of thymosin β4 (TMSB4X) protein in the intima compared with the media layer was found in rabbit and its localization and overexpression in human aortas were confirmed with immunohistochemistry [34, 47]. The previously suggested roles of TMSB4X in tissue regeneration and wound healing and its protective role are in correspondence with the observations.

A MALDI-MSI study into the molecular profiles in human *stenotic aortic valves* showed different peptide patterns for characteristic regions, such as calcification, collagen-rich, elastic fiber-rich areas, and histological layers [56]. Moreover, two peptides involved in the development of calcified aortic stenosis were identified; these peptides come from collagen VI α-3 and N-myc downstream-regulated gene 2 (NDRG-2) protein. Collagen is important in tissue integrity maintenance, suggested to be part of the biomineralization cascade, and calcium accumulation on osteoblast-like cells. NDRG-2 is involved in cell apoptosis, stimulating mineralization. The localization of both proteins around the calcified lesions reinforces their roles.

## Metabolite MSI and cardiovascular diseases

Small molecular compounds (< 1500 Da) are involved in biological and pathological processes; these metabolites are products and intermediates of metabolic pathways. Metabolite studies typically use frozen tissues to prevent degradation; however, Buck et al. demonstrated the use of FFPE for metabolite analysis of human carcinoma tissue samples [14, 61]. The samples were deparaffinized and covered with 9AA matrix followed by MSI in negative ion mode. Alternatively, for the preservation of the chemical composition of a cardiac tissue sample, rapid thermal inactivation is reported to be beneficial [62, 63]. With this technique, fast heating of the sample denatures the enzymes, reducing the degradation of molecular compounds without morphological changes.

The most frequently used *matrices* for metabolites are 9AA and N-(1-naphthyl) ethylenediamine dihydrochloride (NEDC) [63]. For identification purposes, an *instrument* with high mass resolution and accurate mass capabilities is required, for instance, FT-ICR-MS or orbitrap FTMS. Imaging of cardiac metabolites was done using a MALDI-TOF [63] or MALDI Q-TOF instrument [64]. Additionally, peak assignment was done with accurate MS analysis and MS/MS on an ion trap TOF instrument [63].

The majority of the metabolic studies on the heart are gathered using homogenates, losing all spatial information, for example, with LC, GC, or CE-TOF MS [65–67]. In the cardiovascular field, information on the metabolic distributions might provide further insight into pathways involved or the origin of these changes. For example, the metabolite creatine is used for the identification of the *infarcted region* in cardiac tissue; it was previously described as abundant in healthy tissue and reduced in ischemic tissue [28, 36]. As creatine is part of the creatine-phosphocreatine system, these results hint to changes in the energy-related pathways.

**Fig. 4:**
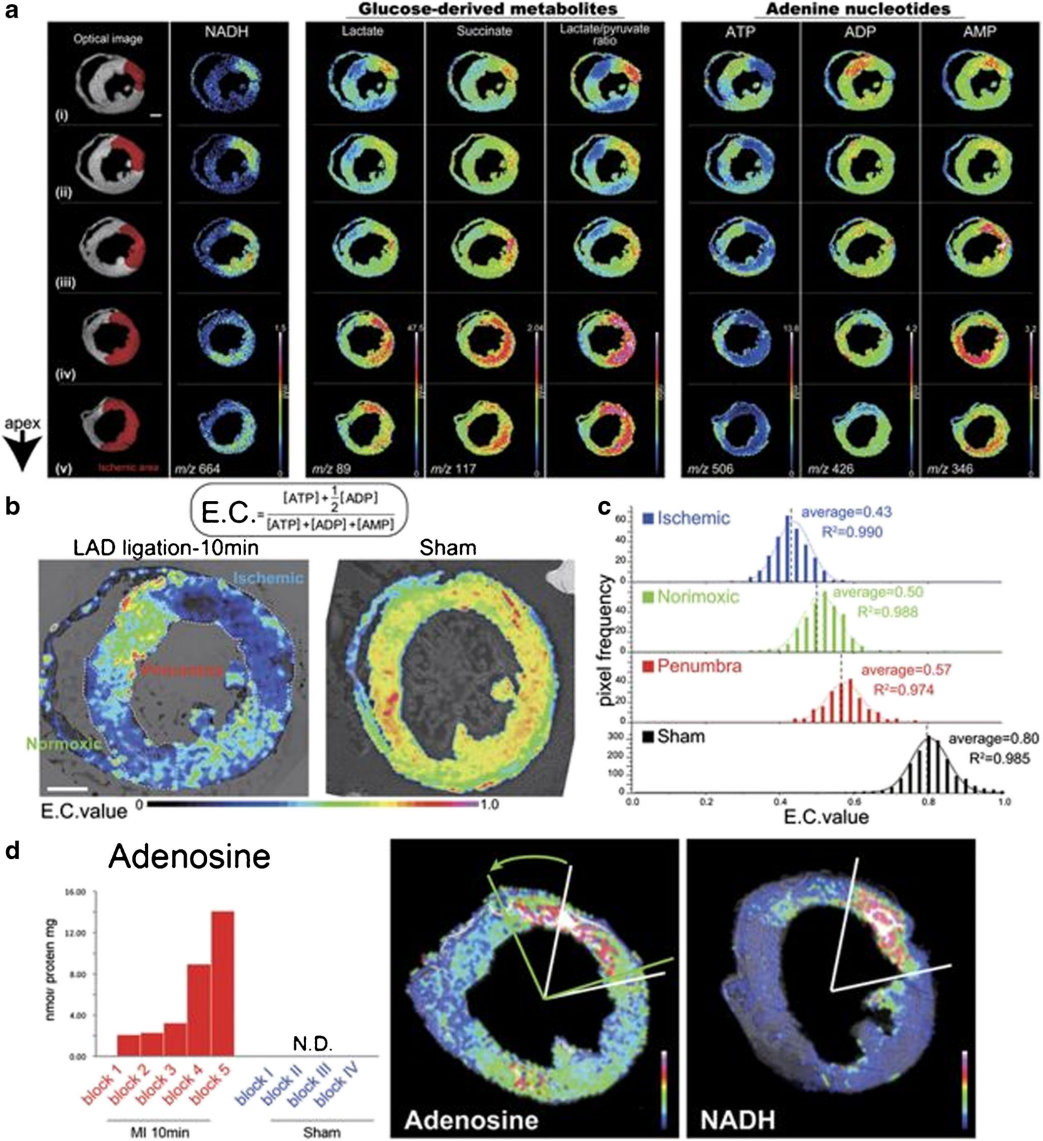
Distribution of cardiac metabolites in an ischemic mouse heart. **a** Optical image of the section indicates the ischemic region in red using NADH as metabolic indicator. The other columns show the ion images of the glucose-derived metabolites and adenine nucleotides as indicated above the images. **b** Energy charge (E.C.) values per pixel were calculated based on MALDI-MSI and normalized using CE-MS-based quantitative analysis. This is presented for the LAD ligated (left) and sham (right) mouse hearts. **c** The E.C. values for the three distinct areas in the LAD ligated heart (ischemic, penumbra, and normoxic regions) and the sham heart. **d** Correlation between adenosine elevation and ischemic severity was detected (left), adenosine overflow in the penumbra region (arrow in the middle panel) from the ischemic core (right panel) was seen. Adapted from Sugiura et al. [63]

By combining MALDI-MSI and capillary electrophoresis (CE)-electrospray ionization (ESI)-MS, Sugiura et al. developed a quantitative MSI protocol [63]. For their analysis of the metabolic dynamics in ischemic mice hearts, focused microwave irradiation was used to minimize postmortem ischemic changes. They distinguished three regions in the infarcted heart with a different metabolite distribution and energy charge (Fig. 4). These ischemic, penumbra, and normoxic regions can be discriminated using NADH, glucose-derived metabolites, and adenine nucleotides.

A multimodal study investigated the role of acute viral myocarditis and inflammation in cardiac metabolic remodeling [64]; they combined imaging with quantitative methods. MALDI-MSI analysis revealed a distinct metabolic profile for viral myocarditis compared with control hearts, the significant decrease of high-energy phosphate, and NAD levels associated with a reduction in the oxidative metabolism of the heart. Also, different metabolic profiles were found within the myocardium distinguishing infiltrated areas from non-infiltrated areas.

## Outlook

Today, MSI approaches are more widely being used for applications in cardiovascular diseases, though this remains limited to research purposes. Most frequently, MALDI-based techniques have been applied with a focus on lipid analysis, including mass spectrometry techniques, to obtain reliable lipid structural information and identification. Moreover, the increasing interest for MSI approaches stimulated the development and optimization of techniques, allowing researchers to use a wider range of (preserved) samples and increase their throughput [68, 69]. Furthermore, to make MSI a diagnostic tool, it is important that standardization, internal standards, quality controls, and normalization strategies are developed and validated by multicenter studies. These are required for a reliable and responsible use.

We also expect that much of the future cardiovascular work will happen in the growing field of “top-down” protein MSI, as it provides a new, comprehensive approach to characterize the cardiac proteome in its native conformation [70]. The application to cardiac proteins to investigate their role in cardiac (dys)function opens up new avenues to treatment and diagnostics for personalized medicine. The combination of spatial information and the relative abundance of cardiac proteins will allow different phases of disease progression. In the future, MSI information will complement the clinical analysis by contributing to the understanding of involved pathways in cardiovascular diseases.

## Electronic supplementary material

ESM 1 (PDF 234 kb)

## Abbreviations

CID: Collision-induced dissociation
DESI: Desorption electrospray ionization
ECD: Electron capture dissociation
FFPE: Formalin-fixed paraffin embedding
FT-ICR: Fourier transform ion cyclotron mass spectrometer
FTMS: Fourier transform mass spectrometry
ITO: Indium tin oxide
LA-ICPMS: Laser ablation inductively coupled mass spectrometry
MS: Mass spectrometry
MSI: Mass spectrometry imaging
MI: Myocardial infarction
MALDI: Matrix-assisted laser desorption/ionization
OCT: Optimal cutting temperature compound
q-TOF: Quadrupole time-of-flight
SIMS: Secondary ion mass spectrometry
TOF: Time-of-flight
UVD: UV photodissociation
CE: Cholesteryl ester
CL: Cardiolipins
FA: Fatty acids
LPC: Lysophosphatidylcholines
PA: Diacylglycerophosphate
PC: Phosphatidylcholines
PE: Phosphatidylethanolamines
PG: Phosphatidylglycerols
PI: Phosphatidylinositols
PL: Phospholipids
PS: Phosphatidylserines
SM: Sphingomyelins
TAG: Triacylglycerols
9AA: 9-Aminoacridine
CHCA: α-Cyano-4 hydroxycinnamic acid
DAN: 1,5-Diaminoaphthalene
DHA: 2,6-Dihydroxyacetophenone
DHB: 2,5-Dihydroxybenzoic acid
NEDC: *N*-(1-Naphthyl)ethylenediamine dihydrochloride
SA: Sinapinic acid
AR: Alizarin red
EVG: Elastica Van Gieson
H&E: Hematoxylin and eosin
IHC: Immunohistochemistry
ORO: Oil Red O
PTAH: Phosphotungstic acid hematoxylin
TTC: 2,3,5-Triphenultetrazolium chloride
VVG: Verhoeff-Van Gieson
