# Supplementary material for: Trends in mass spectrometry imaging for cardiovascular diseases
Source: Anal Bioanal Chem. 2019 Apr 12;411(17):3709–20. doi: 10.1007/s00216-019-01780-8 (PMC6594994; doi:10.1007/s00216-019-01780-8)
Supplement: Supplementary file 1 — (PDF 234 kb) [file 216_2019_1780_MOESM1_ESM.pdf]

Analytical and Bioanalytical Chemistry

Electronic Supplementary Material

Trends in mass spectrometry imaging for cardiovascular diseases

Stephanie T. P. Mezger, Alma M. A. Mingels, Otto Bekers, Berta Cillero-Pastor, Ron M. A. Heeren

Table S1 Overview of MSI research on cardiovascular tissue

		Species	Tissue	Sample preparation	Instrument and settings	Main results	Histology	Ref*
Lipid	MALDI	Rat	Healthy heart	Frozen<br>Washes<br>DHB, sublimation	Autoflex III MALDI-TOF/TOF, Bruker Daltonics<br>Lateral resolution: 100µm<br>Negative ionization mode	Ventricular myocardium: CL (*m/z* 1447.97)	H&E	[1]
		Rat	Healthy heart	Frozen<br>$AgNPs^-$ implantation	MALDI LTQ-XL Orbitrap, Thermo Fisher<br>Lateral resolution: 50µm<br>Positive ionization mode: 600-1100 Da, Negative ionization mode: 600-1700 Da	Vessels region: TAG (+) and PI (-) species<br>Myocardium: PC (+), PE (+&-) and CL (-) species and fragments	n/a	[2]
		Mouse	Healthy heart	Frozen<br>DHB, sublimation	MALDI TOF (homebuilt)<br>Lateral resolution: 9.6µm<br>Positive ionization mode	Hinge: *m/z* 600<br>Hinge and valve cups: *m/z* 741, 846	H&E	[3]
		Rat	LAD ligation model; heart	Frozen<br>DHB with(out) NaOAc, nebulizer	MALDI LTQ-XL Linear ion trap, Thermo Scientific<br>Lateral resolution: 100µm<br>Positive ionization mode, *m/z* 100-250 and *m/z* 200-2000	Infarcted region: lysoPL<br>Perfused region: Intact PL, creatine (*m/z* 132)	TTC	[4]
		Ovine	Healthy adult aortic valves	Frozen<br>Washes<br>DHB, sublimation	UltrafleXtreme MALDI TOF/TOF, Bruker Daltonics<br>Lateral resolution:25µm<br>Positive ionization mode, *m/z* 450-1800	Differentiate fibrosa and spongiosa: PC(12:0/16:0) *m/z* 678.5, SM(d18:1/18:0) *m/z* 731.7, PC(16:0/16:0) *m/z* 732.6, PE(18:1/20:1) *m/z* 774.7, PC(18:0/22:5) *m/z* 836.7	VVG	[5]
			Healthy pre-natal aortic valves			Amorphous valve structure		
		Human	Aortic valve – pediatric donor tissue 0 to 14 years of age – healthy or diseased	OCT – frozen<br>Washes<br>DHB, sublimation	Apex-9.4-Qe FTICR, Bruker Daltonics<br>Spatial resolution: 20µm<br>Positive ionization mode, *m/z* 450-1600	Fibrosa: SM(16:0) *m/z* 703.6<br>Spongiosa: SM(18:0) m/z 731.6	H&E	
		Mouse	$ApoE^{-/-}$ - high cholesterol diet – orally dosed with stable isotope labelled cholesterol<br>Atherosclerotic aortic tissue	OCT – frozen<br>DHB, sublimation or CHCA, acoustic droplet ejection	QTOF MALDI Synapt G2 HDMS, Waters Corporation<br>Spatial resolution: 50µm<br>Positive ionization mode, *m/z* 50-950	Plaque and necrotic core: Free cholesterol (*m/z* 369.3514)<br>Heart tissue: CE (*m/z* 649.5944)<br>Lesion arterial wall and atheroma: LPC species (*m/z* 496.3394, 524.3699, 522.3538)<br>Homogeneous: PC (*m/z* 758.5677) → Lp-LPA2 action	H&E	[6]
		Rabbit	Atherosclerosis model - aortic section	Frozen<br>DHB, sprayed	UltrafleXtreme MALDI-TOF, Bruker Daltonics<br>Spatial resolution: 30µm<br>Dual polarity, 500-1200 Da	Calcified region: PA, SM, and PE-Cer species<br>Increased in intima: SFA, SM, PI, PF, and lysolipid species	H&E, AR<br>ORO<br>IHC	[7]<br>[8]
		Mouse	$ApoE^{-/-}$, aortic sinuses	OCT<br>DAN, sublimation or Silver sputtering	UltrafleXtreme MALDI-TOF/TOF, Bruker Daltonics<br>Spatial resolution: DAN dual polarity, 40µm - Silver-assisted LDI, 30µm	Bulk of plaque: LPC (example: *m/z* 496.3)<br>Plaque: Interior wall - PC species (example: *m/z* 804.5, 832.5), Cholesterol rich regions<br>Aortic valve cusps: TAG species	ORO	[9]
		Human	Atherosclerotic carotid artery	Frozen - OCT<br>DAN, sublimation or Silver sputtering	UltrafleXtreme MALDI-TOF/TOF, Bruker Daltonics<br>Spatial resolution: DAN dual polarity, 100µm - Silver-assisted LDI, 100µm	Plaque: LPC species and ceramide species (*m/z* 616.4, 687.6, and 685.6)	H&E	
		Mouse	$ApoE^{-/-}$, atherosclerotic aortic roots	Paraformaldehyde, CMC<br>DHB, airbrush	Ultraflex II MALDI TOF/TOF, Bruker Daltonics<br>Lateral resolution: 25µm<br>Positive ionization mode, *m/z* 400-1000	Lipid rich: CE species (*m/z* 671.6, 673.6)<br>Smooth muscle cells: PC species (m/z 804.5, 832.5)<br>Calcified region: *m/z* 566.9	H&E,<br>ORO<br>IHC	[10]
		Human	Atherosclerotic femoral artery	Frozen<br>DHB, airbrush	Ultraflex II MALDI TOF/TOF, Bruker Daltonics<br>Lateral resolution: 50µm<br>Positive ionization mode, *m/z* 400-1000	Lipid rich: CE (*m/z* 671.6, 673.6) and TAG species (*m/z* 907.7)<br>Smooth muscle cells: PC species (*m/z* 804.5, 832.5)<br>Calcified region: *m/z* 539.0		
		Mouse	$ApoE^{-/-}$, aortic sample	Frozen	Ultraflex II MALDI TOF/TOF, Bruker Daltonics	Atherosclerotic tissue: increase LPC and decrease	H&E,	[11]

		Human	Atherosclerotic femoral artery	DHB, airbrush	Lateral resolution: 50µm<br>Positive ionization mode, *m/z* 400-1000	arachidonyl-PC<br>Arachidonyl-PC region: increase LPCAT3 expression	EVG<br>IHC	
LIPID	SIIMS	Mouse	Healthy heart	OCT - frozen	LA (New wave, Fremont, US) coupled to ICPMS (quadrupole, XSeries2), Thermo Fischer Scientific<br>Spatial resolution: 160µm<br>TOF SIMS IV, IONTOF GmbH - Bismuth liquid metal ion gun<br>Lateral resolution: 33µm, *m/z* up to 800	Right ventricle: higher Zn, Mn, Cu, Mg, and Ca<br>Aorta: higher Fe<br>Endocardium: choline and its fragment (*m/z* 104.21, 84.12), cholesterol fragment (*m/z* 146.97)	H&E	[12]
		Rat	Healthy aorta	Frozen, freeze-fracture, free-dried	TOF SIMS IV & V, IONTOF GmbH<br>Bismuth liquid metal ion gun<br>Ion beam focus: 300nm<br>Dual polarity	Intima and media (between lamellae): choline headgroup (*m/z* 184); Media (lamellae region): potassium, cholesterol (*m/z* 369, 385)	Toluidine blue/azan	[13]
		Human	Atherosclerotic plaque	Frozen		Intima: cholesterol (spots), PC and SM species<br>Media: PC and DAG species	n/a	
		Rat	Healthy heart	Frozen<br>Metallization: gold sputtered	TOF SIMS TRIFT II, Physical Electronics,<br>Gold liquid metal ion gun<br>Positive ionization mode, *m/z* up to 1500	Aorta wall (*m/z* 667 and 840), Pericardium (*m/z* 334), Ventricles (*m/z* 175 and 213)<br>Aorta wall, semilunar valve, endocardium: *m/z* 83<br>Pulmonary artery, right atrium, atrioventricular valve: *m/z* 145; Atria, aorta wall, atrioventricular valves, coronary artery: cholesterol (*m/z* 369, 385); Atria, aortic wall, aorta valve: *m/z* 86; Atria, aorta, pulmonary artery, atrioventricular and semilunar valves: choline (*m/z* 104)	H&E	[14]
		Mouse	Healthy heart			Mouse similar patterns as rat		
		Human	Ventricle from failing left-ventricular free-wall heart explants			Myocardium: cholesterol choline (*m/z* 369), choline (*m/z* 104); Endocardium: choline (*m/z* 104); Pericardium: DAG species (*m/z* 549, 557) and ceramide (*m/z* 604)	H&E<br>Sirius Red<br>Trichrome	
		Mouse	$LDLr^{-/-}$ model; Atherosclerotic carotid artery	Gelatin – Frozen	TOF-SIMS TRIFT II, Physical Electronics<br>Gold liquid metal ion gun<br>Dual polarity	Necrotic core: LPA species, cholesterol, phosphatidic acids, triglycerides	H&E<br>IHC	[15]
		Rat	Healthy heart	Frozen<br>Silicon substrate	TOF SIMS IV, IONTOF, Gold liquid metal ion gun<br>Lateral resolution: ~3.9µm<br>Dual polarity	Complementary distribution of PL and CL species, 16 and 18-C FA species	n/a	[16]
			Healthy left ventricular cells	Isolated cells<br>25µl on silicon substrate	TOF SIMS IV, IONTOF, Gold liquid metal ion gun<br>Lateral resolution: ~430nm<br>Positive ionization mode	Cell membrane: phospholipids and cholesterol species		
		Human	Atherosclerotic carotid artery	Frozen	TOF-SIMS IV, IONTOF, Bismuth cluster ion source<br>Spatial resolution: 1-2µm<br>Dual polarity, *m/z* 1-1000	Inner border: cholesterol (*m/z* 385), phosphatidic acids (*m/z* 650-750) and SM fragments (*m/z* 616, 642)<br>Surrounding and thrombus: vitamin E (*m/z* 430)<br>Intima: PI fragments	Masson Trichrome	[17]
LIPID	DESI	Human	Atherosclerotic carotid artery	Frozen<br>CMC for sectioning	DESI stage (lab built) coupled to LTQ (Thermo Scientific) - Normal & Reactive DESI<br>Spatial resolution: 200µm<br>Positive ionization mode, *m/z* 150-1200	Plaque: SM and PC species, and cholesterol<br>Lipid core: CE species surrounded by cholesterol, PC, and SM	H&E	[18]
		Mouse	LAD ligation model; heart	Frozen	DESI stage (custom built) LTQ-Orbitrap XL (Thermo Fisher Scientific)<br>Lateral resolution: 0,2mm<br>Negative ionization mode, *m/z* 50-1200	Infarcted tissue: FA(18:1) *m/z* 281.248, FA(18:0) *m/z* 283.264, FA(16:1) *m/z* 253.217, and FA(16:0) *m/z* 255.232<br>Perfused tissue: glycerophospholipid species, polyunsaturated FAs, and taurine (*m/z* 124.007)<br>GBDT algorithm: 62 peaks for classification	H&E	[19]

		Human	Vascular graft	Frozen	DESI source (OMNIspray) AmaZon ETD (Bruker Daltonics) - Normal & Reactive DESI<br>Dual polarity, *m/z* 300-1200	Graft: SM, PC species and cholesterol. Graft Wall: SM (24:1), Biofilm on graft wall: PS (38:4), Plaque: cholesterol	n/a	[20]
Peptide/protein	MALDI	Chicken	Healthy heart	Fixation - Embedding<br>Dewaxing<br>Washes<br>DHB, CHCA or SA, nebulizer	Autoflex III MALDI TOF/TOF, Bruker Daltonics, Linear mode<br>Lateral resolution: 75-100µm<br>Positive ionization mode, *m/z* 3000-30000	Myocardium (*m/z* 9492, 11862), Myocardium right ventricle (*m/z* 6614), Atrial myocardium (*m/z* 6643)<br>Outer ventricular wall (*m/z* 6643), Walls major heart vessels layers (*m/z* 7475, 6143, and 11862)<br>Aortic valvular tissue (*m/z* 5308,) Aortic intimal and adventitial tissue (*m/z* 5935)<br>Apex interventricular septum (*m/z* 6669), Bundle of His (*m/z* 9329), Valve structures (*m/z* 12209), Leaflets left AV valve (*m/z* 3915)	n/a	[21]
		Mouse	LAD ligation model; heart	Frozen<br>ANG II	Autoflex III MALDI TOF/TOF, Bruker Daltonics	Infarct area: ANG III (*m/z* 931) and ANG-(2-7) (*m/z* 784)<br>Increase of APA in MI tissue	TTC	[22]
		Mouse	LAD ligation model; heart	Frozen<br>Washes<br>Intact: SA, TM sprayer<br>Peptide: trypsin, SA, TM sprayer	Autoflex Speed MALDI-TOF/TOF, Bruker Daltonics<br>Spatial distribution: 100µm<br>Positive ionization mode<br>Intact: linear mode<br>Peptides: reflectron mode	Healthy cardiac tissue: creatine (*m/z* 132)<br>Remote: altered enzymatic activity<br>Border: high transcription and translation Infarcted: mitochondrial and metabolic enzymes	H&E	[23]
			Healthy heart			Healthy cardiac tissue: *m/z* 908, 1632.96, 2816.25, and 4497.11 (Ephrin A1 peptides)		
		Mouse<br>Human	MI heart samples	FFPE<br>Pre-treatment buffer<br>Trypsin, DHB, chemical inkjet printer	MALDI TOF/TOF (AXIMA Performance and 7090 series; Shimadzu)<br>Positive ionization mode, *m/z* 700-3000	Endocardium: Haemoglobin subunit α (*m/z* 1529.997)<br>Infarcted region: MYH6, MYL3, ATP5A, MYH7, and ACTA2 (*m/z* 1084.64, 1396.96, 1625.09, 1741.12, and 1956.24, respectively)	H&E<br>PTAH<br>IHC	[24]
		Rabbit	Atherosclerosis model - aortic section	Frozen<br>Washes<br>SA, sprayed	UltrafleXtreme MALDI-TOF, Bruker Daltonics<br>Spatial resolution: 30µm<br>Linear positive, 2000-20000 kDa	TMSB4X (*m/z* 4762) upregulated	IHC	[7]<br>[8]
		Human	Heart valve with moderate CAS	Frozen<br>Carnoy procedure<br>SA, sprayed	Autoflex III smartbeam MALDI-TOF/TOF, Bruker Daltonics<br>Spatial resolution: 75µm<br>Positive ionization mode, 1000-30000Da	Calcified area (5059 Da), Collagen-rich (4300 Da), Elastic fibers-rich (13984 Da), Surrounding tissue (14659 Da), Margins calcified area: NDRG-2 (peptide, 3398 Da), Around calcified lesion: CO6A3 (peptide, 4321 Da)	VVG<br>ORO<br>IHC	[25]
		Human	Left atrial appendage specimens<br>Atrial fibrillation (PX, PE, and LSP)	FFPE<br>Dewaxing, Washes, Antigen retrieval<br>Trypsin, CHCA, Image Prep	Autoflex III MALDI TOF/TOF, Bruker Daltonics<br>Lateral resolution: 80µm<br>Positive ionization mode, *m/z* 800-3500	Decreased in PX: ATPA (*m/z* 1000.489), MYL4 (*m/z* 1262.601), H13 (*m/z* 1260.618), AHNK (*m/z* 901.472), CDH13 (*m/z* 1564.840), VIM (*m/z* 1093.555)<br>Increased in LSP and PE: CO1A1 (*m/z* 837.393)	IHC	[26]
Metabolites	MALDI	Mouse	LAD ligation model; heart<br><br>13C labelled glucose & lactate injection	FMW fixation<br>Super cryo embedding medium<br>9AA, manual spray coated	UltrafleXtreme MALDI TOF, Bruker Daltonics<br>Lateral resolution: 100µm<br>Negative ionization mode, *m/z* 50-1000	Ischemic core: increase NADH, lactate, succinate, $^{13}C_3$-glutamate; Non-ischemic region adjacent to core: ATP, ADP; More distant region: higher lactate/pyruvate ratio	n/a	[27]
		Mouse	C3H mice<br>- injected with CVB3 virus (VM hearts)<br>- control	Frozen<br>9AA, Suncollect	QTOF MALDI SYNAPT HDMS G2Si, Waters<br>Lateral resolution: 100µm<br>Negative ionization mode	VM hearts vs control: decrease ATP, ADP, AMP, total adenine nucleotide, NAD<br>Increase UDP-GlcNAc, PI, AA, cardiolipin	H&E	[28]

* Reference as listed below. Not equal to the main manuscript
Reflectron mode unless stated otherwise, +: positive ionization mode, -: negative ionization mode
AR: Alizarin Red, EVG: Elastica van Gieson, H&E: Haematoxylin and eosin, IHC: Immunohistochemistry, ORO: Oil Red O, PTAH: Phosphotungstic acid-haematoxylin, TTC: 2,3,5-triphenultetrazolium chloride, VVG: Verhoeff-Van Gieson

References

1. Wang HY, Wu HW, Tsai PJ, Liu CB, Zheng ZF. Matrix-assisted laser desorption/ionization mass spectrometry imaging of cardiolipins in rat organ sections. Anal Bioanal Chem. 2014;406(2):565-75. doi:10.1007/s00216-013-7492-y.
2. Jackson SN, Baldwin K, Muller L, Womack VM, Schultz JA, Balaban C et al. Imaging of lipids in rat heart by MALDI-MS with silver nanoparticles. Anal Bioanal Chem. 2014;406(5):1377-86. doi:10.1007/s00216-013-7525-6.
3. Chaurand P, Cornett DS, Angel PM, Caprioli RM. From whole-body sections down to cellular level, multiscale imaging of phospholipids by MALDI mass spectrometry. Mol Cell Proteomics. 2011;10(2):O110 004259. doi:10.1074/mcp.O110.004259.
4. Menger RF, Stutts WL, Anbukumar DS, Bowden JA, Ford DA, Yost RA. MALDI mass spectrometric imaging of cardiac tissue following myocardial infarction in a rat coronary artery ligation model. Anal Chem. 2012;84(2):1117-25. doi:10.1021/ac202779h.
5. Angel PM, Bayoumi AS, Hinton RB, Ru Su Y, Bichell D, Mayer JE et al. MALDI Imaging Mass Spectrometry as a Lipidomic Approach to Heart Valve Research. J Heart Valve Dis. 2016;25(2):240-52.
6. Castro-Perez J, Hatcher N, Kofi Karikari N, Wang SP, Mendoza V, Shion H et al. In vivo isotopically labeled atherosclerotic aorta plaques in ApoE KO mice and molecular profiling by matrix-assisted laser desorption/ionization mass spectrometric imaging. Rapid Commun Mass Spectrom. 2014;28(22):2471-9. doi:10.1002/rcm.7039.
7. Martin-Lorenzo M, Balluff B, Maroto AS, Carreira RJ, van Zeijl RJ, Gonzalez-Calero L et al. Molecular anatomy of ascending aorta in atherosclerosis by MS Imaging: Specific lipid and protein patterns reflect pathology. J Proteomics. 2015;126:245-51. doi:10.1016/j.jprot.2015.06.005.
8. Martin-Lorenzo M, Balluff B, Maroto AS, Carreira RJ, van Zeijl RJ, Gonzalez-Calero L et al. Lipid and protein maps defining arterial layers in atherosclerotic aorta. Data Brief. 2015;4:328-31. doi:10.1016/j.dib.2015.06.005.
9. Patterson NH, Doonan RJ, Daskalopoulou SS, Dufresne M, Lenglet S, Montecucco F et al. Three-dimensional imaging MS of lipids in atherosclerotic plaques: Open-source methods for reconstruction and analysis. Proteomics. 2016;16(11-12):1642-51. doi:10.1002/pmic.201500490.
10. Zaima N, Sasaki T, Tanaka H, Cheng XW, Onoue K, Hayasaka T et al. Imaging mass spectrometry-based histopathologic examination of atherosclerotic lesions. Atherosclerosis. 2011;217(2):427-32. doi:10.1016/j.atherosclerosis.2011.03.044.
11. Tanaka H, Zaima N, Sasaki T, Yamamoto N, Inuzuka K, Yata T et al. Lysophosphatidylcholine Acyltransferase-3 Expression Is Associated with Atherosclerosis Progression. J Vasc Res. 2017;54(4):200-8. doi:10.1159/000473879.
12. Becker JS, Breuer U, Hsieh HF, Osterholt T, Kumtabtim U, Wu B et al. Bioimaging of metals and biomolecules in mouse heart by laser ablation inductively coupled plasma mass spectrometry and secondary ion mass spectrometry. Anal Chem. 2010;82(22):9528-33. doi:10.1021/ac102256q.
13. Malmberg P, Borner K, Chen Y, Friberg P, Hagenhoff B, Mansson JE et al. Localization of lipids in the aortic wall with imaging TOF-SIMS. Biochim Biophys Acta. 2007;1771(2):185-95. doi:10.1016/j.bbalip.2006.12.003.
14. Fornai L, Angelini A, Klinkert I, Giskes F, Kiss A, Eijkel G et al. Three-dimensional molecular reconstruction of rat heart with mass spectrometry imaging. Anal Bioanal Chem. 2012;404(10):2927-38. doi:10.1007/s00216-012-6451-3.
15. Bot M, de Jager SC, MacAleese L, Lagraauw HM, van Berkel TJ, Quax PH et al. Lysophosphatidic acid triggers mast cell-driven atherosclerotic plaque destabilization by increasing vascular inflammation. J Lipid Res. 2013;54(5):1265-74. doi:10.1194/jlr.M032862.
16. Jerigova M, Biro C, Kirchnerova J, Chorvatova A, Chorvat D, Jr., Lorenc D et al. Chemical imaging of cardiac cell and tissue by using secondary ion mass spectrometry. Mol Imaging Biol. 2011;13(6):1067-76. doi:10.1007/s11307-010-0460-4.
17. Mas S, Touboul D, Brunelle A, Aragoncillo P, Egido J, Laprevote O et al. Lipid cartography of atherosclerotic plaque by cluster-TOF-SIMS imaging. Analyst. 2007;132(1):24-6. doi:10.1039/b614619h.

18. Manicke NE, Nefliu M, Wu C, Woods JW, Reiser V, Hendrickson RC et al. Imaging of lipids in atheroma by desorption electrospray ionization mass spectrometry. Anal Chem. 2009;81(21):8702-7. doi:10.1021/ac901739s.
19. Margulis K, Zhou Z, Fang Q, Sievers RE, Lee RJ, Zare RN. Combining Desorption Electrospray Ionization Mass Spectrometry Imaging and Machine Learning for Molecular Recognition of Myocardial Infarction. Anal Chem. 2018. doi:10.1021/acs.analchem.8b03410.
20. Bodzon-Kulakowska A, Drabik A, Mystkowska J, Chlabicz M, Gacko M, Dabrowski JR et al. Desorption electrospray ionization-based imaging of interaction between vascular graft and human body. J Biomed Mater Res B Appl Biomater. 2016;104(1):192-6. doi:10.1002/jbm.b.33385.
21. Grey AC, Gelasco AK, Section J, Moreno-Rodriguez RA, Krug EL, Schey KL. Molecular morphology of the chick heart visualized by MALDI imaging mass spectrometry. Anat Rec (Hoboken). 2010;293(5):821-8. doi:10.1002/ar.21103.
22. Alghamri MS, Morris M, Meszaros JG, Elased KM, Grobe N. Novel role of aminopeptidase-A in angiotensin-(1-7) metabolism post myocardial infarction. Am J Physiol Heart Circ Physiol. 2014;306(7):H1032-40. doi:10.1152/ajpheart.00911.2013.
23. Lefcoski S, Kew K, Reece S, Torres MJ, Parks J, Reece S et al. Anatomical-Molecular Distribution of EphrinA1 in Infarcted Mouse Heart Using MALDI Mass Spectrometry Imaging. J Am Soc Mass Spectrom. 2018;29(3):527-34. doi:10.1007/s13361-017-1869-7.
24. Yajima Y, Hiratsuka T, Kakimoto Y, Ogawa S, Shima K, Yamazaki Y et al. Region of Interest analysis using mass spectrometry imaging of mitochondrial and sarcomeric proteins in acute cardiac infarction tissue. Sci Rep. 2018;8(1):7493. doi:10.1038/s41598-018-25817-7.
25. Mourino-Alvarez L, Iloro I, de la Cuesta F, Azkargorta M, Sastre-Oliva T, Escobes I et al. MALDI-Imaging Mass Spectrometry: a step forward in the anatomopathological characterization of stenotic aortic valve tissue. Sci Rep. 2016;6:27106. doi:10.1038/srep27106.
26. Klein O, Hanke T, Nebrich G, Yan J, Schubert B, Giavalisco P et al. Imaging Mass Spectrometry for Characterization of Atrial Fibrillation Subtypes. Proteomics Clin Appl. 2018:e1700155. doi:10.1002/prca.201700155.
27. Sugiura Y, Katsumata Y, Sano M, Honda K, Kajimura M, Fukuda K et al. Visualization of in vivo metabolic flows reveals accelerated utilization of glucose and lactate in penumbra of ischemic heart. Sci Rep. 2016;6:32361. doi:10.1038/srep32361.
28. Remels AHV, Derks WJA, Cillero-Pastor B, Verhees KJP, Kelders MC, Heggermont W et al. NF-kappaB-mediated metabolic remodelling in the inflamed heart in acute viral myocarditis. Biochim Biophys Acta. 2018;1864(8):2579-89. doi:10.1016/j.bbadis.2018.04.022.
